# Predicting bleb revision after XEN gel stent surgery in a real-world open-angle glaucoma cohort

**DOI:** 10.1007/s00417-026-07255-8

**Published:** 2026-04-25

**Authors:** Caroline J. Wenzel, Christina Pagonidou, Daniel A. Wenzel, Vasyl Druchkiv, Emil Nasyrov, Bogomil Voykov

**Affiliations:** 1 Department of Ophthalmology, University Hospital Tuebingen, Tuebingen, Germany; 2 Augencentrum Südwest, Stuttgart, Germany; 3 Department of Research and Development, Clinica Baviera-AIER Eye Group, Valencia, Spain; 4 Department of Ophthalmology, University Clinic Hamburg-Eppendorf, University Medical Center Hamburg-Eppendorf, Hamburg, Germany; 5 Centre for Ophthalmology, University Hospital Tuebingen, Elfriede-Aulhorn-Str. 7, Tübingen, 72076 Germany

**Keywords:** XEN gel stent, Bleb revision, Needling, Bleb fibrosis, Glaucoma surgery, Less invasive bleb surgery, Minimally invasive bleb surgery

## Abstract

**Purpose:**

To evaluate the incidence, timing, and risk factors for postoperative needling procedure and incisional bleb revision following XEN gel stent implantation in a large single-center cohort with a long-term follow-up.

**Methods:**

This retrospective observational cohort study included 773 eyes from 596 patients who underwent XEN gel stent implantation between December 2015 and July 2023. Patients had primary or secondary open-angle glaucoma and at least 12 months of follow-up. Primary outcomes were the need for surgical bleb revision (needling procedure or incisional bleb revision). Secondary outcomes included timing and frequency of interventions. Cox regression models were used to identify risk factors.

**Results:**

A total of 417 eyes (53.9%) underwent at least one needling procedure, and 137 eyes (17.8%) required incisional bleb revision. Most interventions occurred within the first 100 weeks. Mean time to first needling procedure was 33.6 ± 49.1 weeks, and to first incisional revision 60.9 ± 62.5 weeks. The mean interval between needling procedure followed by incisional revision was 31.8 weeks, and 22.2 weeks for the reverse sequence. Cox regression revealed that male sex (HR 1.43; 95% CI, 1.03–1.99; p = 0.035) and phakic lens status (HR 0.59 for pseudophakia; 95% CI, 0.42–0.82; p = 0.002) were independently associated with incisional bleb revision. No examined baseline variables predicted the need for needling procedures.

**Conclusion:**

Postoperative bleb interventions are common after XEN gel stent implantation, occurring most frequently within the first two years but also extending into long-term follow-up. Male sex and phakic lens status are associated with a higher risk of incisional bleb revision, while needling procedure appears less dependent on baseline characteristics. These findings may help refine patient counselling, risk stratification, and postoperative management strategies.

## Key message

What is known

- Subconjunctival fibrosis is a common cause of long-term failure after XEN gel stent implantation and frequently requires postoperative blebre-intervention to restore aqueous outflow.

What is new

- In a large real-world cohort with long follow-up, postoperative bleb interventions after XEN implantation were frequent, with more than half of eyes requiring needling procedure and nearly one in five undergoing incisional bleb revision, mainly within the first two years but also at later stages.
- Male sex and phakic lens status independently increased the risk of incisional bleb revision, whereas no baseline clinical factors predicted the need for needling procedure.

## Introduction

Elevated intraocular pressure (IOP) is the most significant modifiable risk factor in glaucoma progression, making its reduction the central goal of treatment [1]. When pharmacological therapy fails to achieve adequate IOP control or is associated with intolerable side effects, surgical interventions become necessary to prevent further optic nerve damage. Traditional filtration surgery, such as trabeculectomy, is effective but carries a risk of complications and prolonged recovery [2]. The XEN gel stent (AbbVie Inc., North Chicago, Illinois, USA), a minimally invasive bleb surgery (MIBS) device, was developed to offer a less invasive alternative with shorter rehabilitation periods. It is a 6 mm long tube made of porcine gelatine crosslinked with glutaraldehyde [3, 4], lowering IOP by diverting aqueous humor into the subconjunctival space and forming a filtering bleb. The XEN gel stent is implanted via an ab interno approach, avoiding conjunctival dissection and reducing surgical trauma compared to conventional procedures [5]. To minimize postoperative fibrosis, mitomycin C (MMC) is routinely applied intraoperatively [6]. Despite these advantages, the long-term success of the XEN gel stent – like all bleb-forming procedures – remains limited by subconjunctival fibrosis, leading to bleb failure and requiring postoperative interventions to restore outflow. Needling procedure is a minimally invasive postoperative intervention, as it can be performed without opening the conjunctiva, whereas incisional bleb revision involves surgical dissection to re-establish filtration.

This study aimed to identify potential predictors of postoperative needling procedure and incisional bleb revision, and to evaluate the incidence and timing of bleb revision surgery in a large single-center cohort of patients undergoing XEN45 gel stent implantation, thereby providing a more comprehensive understanding of postoperative intervention patterns. Knowledge of such risk factors contributing to the failure of bleb function can facilitate patient selection for this surgical method, improve counselling of the individual patient and help refining surgical strategies.

## Methods

### Study design

This study is a large retrospective, observational single-center cohort analysis. All XEN45 gel stent implantations and subsequent surgical bleb revisions were performed by a single experienced surgeon.

Patients were eligible for inclusion if they had been diagnosed with primary or secondary open-angle glaucoma, had insufficient IOP control despite maximally tolerated medical therapy and underwent XEN gel stent implantation between December 2015 and July 2023. Only eyes with a follow-up period of at least 12 months were considered. Exclusion criteria were primary angle-closure glaucoma, insufficient follow-up data and implantation of the XEN63 gel stent.

This study adhered to the tenets of the Declaration of Helsinki. All patients provided written informed consent prior to surgery. The requirement for additional written consent for retrospective study participation was waived by the institutional ethics committee. Ethical approval was obtained from the institutional ethics board of the University of Tuebingen (project number: 423/2021B02).

### Outcome measures

The primary outcome of this study was the identification of clinical and demographic factors associated with postoperative needling procedure and incisional bleb revision, following XEN45 gel stent implantation.

Secondary outcome measures were the time to first needling procedure or incisional bleb revision after XEN gel stent implantation, the number of needling procedures and incisional bleb revisions, and the intraoperative use of antimetabolites during revision procedures.

### Surgical technique

### Needling procedure

Needling procedure was performed under topical anesthesia with oxybuprocaine eye drops following previously described methods [7]. A bent 30G needle was used to inject a small subconjunctival bubble of mepivacaine next to the XEN gel stent. Fibrotic tissue around the stent was then gently disrupted with sweeping needle movements, and 5 µg MMC (0.025 mL of 0.2 mg/mL) was injected adjacent to the bleb. When 5-fluorouracil (5-FU) was used, 100 µl of 5-FU (50 mg/ml; total dose 5 mg) was injected subconjunctivally adjacent to the bleb.

### Incisional bleb revision

Incisional bleb revision was performed under parabulbar anesthesia as previously described [8]. A traction suture was placed in the superior peripheral cornea. After two radial incisions of the conjunctiva, the subconjunctival space was bluntly dissected and scar tissue was removed. Two sponges soaked with 0.2 mg/mL MMC were placed under the conjunctiva for 3 min. The area was subsequently irrigated with balanced salt solution. After confirming aqueous outflow through the stent, the conjunctiva was closed with 10 − 0 nylon sutures. Subconjunctival injections of dexamethasone and gentamicin were given at the end of the procedure.

### Pre- and postoperative management

After needling procedure or incisional bleb revision, patients received moxifloxacin eye drops four times daily for three days, and preservative-free dexamethasone eye drops according to a 5-week taper, starting with five applications on postoperative day one.

Postoperative examinations were conducted on the first two days after surgery, followed by visits at 3, 6, 9, and 12 months, and thereafter at six-month intervals up to a maximum of 90 months depending on patient availability.

If IOP was inadequately controlled or clinical signs of bleb scarring were observed, a needling procedure or incisional bleb revision was performed rather than initiating topical IOP-lowering medication. A needling procedure was generally preferred if the XEN gel stent was clearly visible beneath the conjunctiva. When the stent was not visible under the conjunctiva or the degree of fibrosis suggested that disruption through a transconjunctival approach would be ineffective, an incisional bleb revision was carried out as previously described. Final decisions were made at the surgeon’s discretion.

### Statistical analysis

Descriptive statistics were used to summarize demographic and clinical characteristics. Kaplan–Meier survival curves were generated for categorical variables. For continuous predictors, Kaplan–Meier curves were plotted after categorising values into quantile-based groups using the 25th and 75th percentiles (Q1, Q3): low (< Q1), middle (Q1–Q3), and high (> Q3). These visualisations are exploratory and intended to illustrate unadjusted survival patterns, complementing multivariable Cox regression analyses. Potential risk factors associated with surgical success were assessed using multivariate logistic regression.

Data were reported as frequency (percentage), mean (standard deviation [SD]), mean (95% confidence interval [95% CI]), or median (range), as appropriate. A p-value of < 0.05 was considered statistically significant.

## Results

### Characteristics of the study population

A total of 773 eyes of 596 patients who underwent XEN gel stent implantation were included in the study. The mean (± SD) age was 66 (± 16) years. 421 eyes (54.5%) were from male patients, and 352 eyes (45.5%) were from female patients.

At the time of XEN gel stent implantation, 388 eyes (50.2%) were phakic, whereas 385 eyes (49.8%) were pseudophakic. The majority of eyes (405 eyes, 52.4%) had no history of prior glaucoma surgery or laser treatment. In 90 eyes (11.6%), XEN implantation was combined with cataract surgery.

Baseline patient characteristics are summarized in Table 1.

The majority of eyes (*n* = 431, 55.8%) had primary open-angle glaucoma (POAG), whereas 160 (20.7%) had pseudoexfoliative glaucoma, 65 (8.3%) had uveitic glaucoma, 44 (5.7%) pigmentary glaucoma, 20 (2.6%) normal tension glaucoma, 20 (2.6%) congenital glaucoma, 15 (1.9%) neovascular glaucoma, 8 (1.0%) traumatic glaucoma, 4 (0.5%) aphakic glaucoma, and 2 eyes (0.3%) each had glaucoma secondary to iridocorneal endothelial syndrome, iris melanoma, and uveitis-glaucoma-hyphema syndrome.

**Table 1:** Baseline demographic and clinical characteristics of the study patients

Demographic and clinical characteristics of the study population		
Characteristic	*N*	%
Number of eyes (right/left)	773 (364/409)	100 (47.1/52.9)
Age in years, mean ± SD	66.0 ± 16.0	
Gender (male/female)	421/352	54.5/45.5
Lens status (phakic/pseudophakic)	388/385	49.8/50.2
**Prior glaucoma surgery or laser treatment**		
**Procedure**	***N***	**%**
Selective laser trabeculoplasty	78	10.1
Diode laser cyclophotocoagulation	105	13.6
Trabeculectomy	69	8.9
Canaloplasty	21	2.7
Deep sclerectomy	3	0.4
Trabeculotomy	46	5.9
Ahmed glaucoma valve	14	1.8
Cyclocryocoagulation	32	4.1

### Risk factors for the need for incisional bleb revision

A Cox regression model was used to assess whether the hazard ratio for the need for incisional bleb revision was significantly associated with one of the following factors: age, sex, baseline IOP, IOP on the first postoperative day, lens status (phakic/pseudophakic), and previous incisional glaucoma surgery. To exclude potential collinearity among the investigated factors, we applied a backward selection algorithm to the saturated Cox model, including all main effects and interactions, to identify the variables that were independently relevant.

The analysis revealed that sex and lens status showed a statistically significant association with the risk of incisional bleb revision.

The results indicate that the hazard for requiring bleb revision was higher in male patients and lower in pseudophakic eyes. Specifically, the estimated hazard ratio for male sex was 1.430 (95% CI [1.025, 1.996], *p* = 0.035), suggesting a 43% increased risk compared to female patients. Conversely, pseudophakic eyes exhibited a significantly lower risk, with a hazard ratio of 0.587 (95% CI [0.421, 0.818], *p* = 0.002), indicating a 41.3% reduction in the likelihood of incisional bleb revision compared to phakic eyes.

**Fig. 1:**
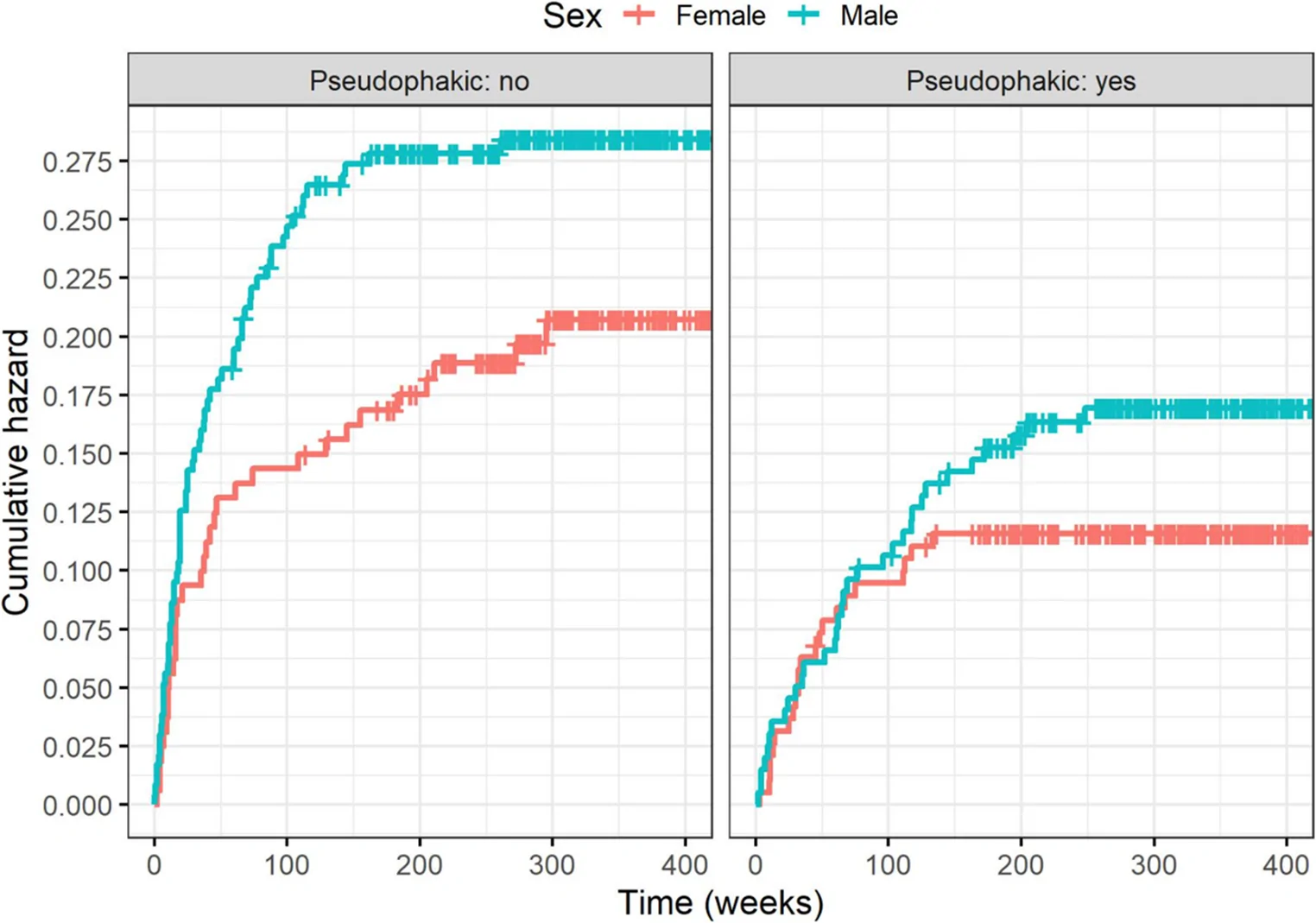
Kaplan-Meier curves showing the cumulative hazard of requiring incisional bleb revision. Phakic eyes are depicted in the left, pseudophakic eyes in the right graph. Blue lines represent eyes from male patients, red lines those from female patients. Male patients and phakic eyes presented significantly higher hazard ratios of requiring incisional bleb revision

Kaplan-Meier curves were plotted for sex and lens status (Fig. 1).

### Risk factors for the need of needling procedure

The Cox regression model revealed that the hazard ratio for the need for needling procedure was not significantly associated with any of the following factors: age, sex, baseline IOP, IOP on the first postoperative day, lens status, and previous incisional glaucoma surgery.

### Incidence and timing of postoperative bleb interventions

A needling procedure was performed at least once in 417 (53.9%) of the studied eyes, and an incisional bleb revision was necessary at least once in 137 (17.8%) of the eyes. A mean (± SD, median) and median of 0.86 (± 0.99 SD, median: 1) needling procedures and 0.20 (± 0.46 SD, median: 0) incisional bleb revisions per eye were performed across all eyes included in this study.

The mean time to the first needling procedure was 33.6 weeks (± 49.1 weeks SD, median: 14 weeks), while the mean time to the first incisional bleb revision was 60.9 weeks (± 62.5 weeks SD, median: 37 weeks).

**Fig. 2:**
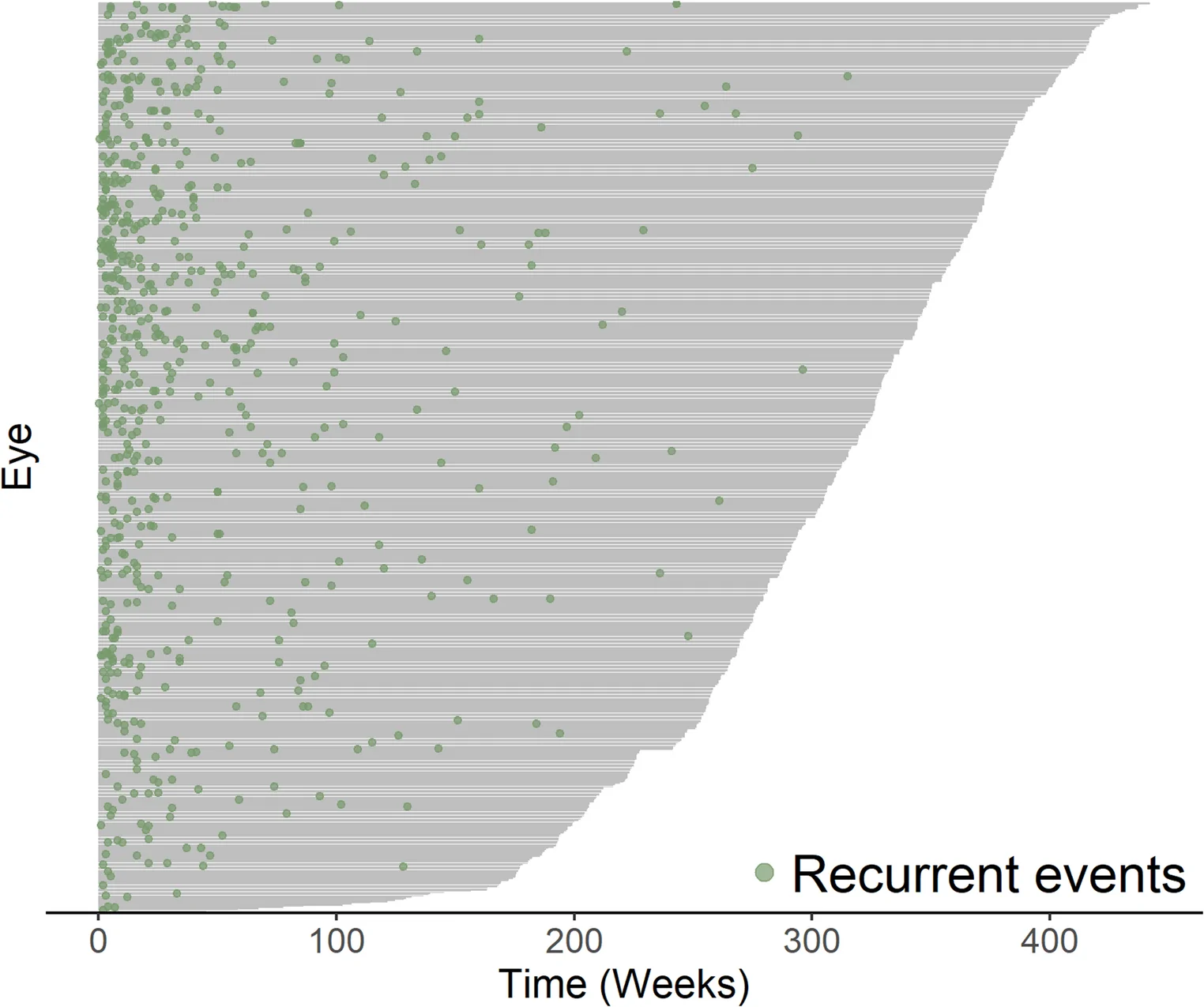
Timing of postoperative needling procedures following XEN45 gel stent implantation. Each green dot represents one needling procedure. The majority of needling procedures occured early after surgery, predominantly within the first 100 weeks (∼ 2 years), illustrating the temporal distribution of needling procedures in this cohort

The temporal distribution of postoperative needling procedures is shown in Fig. 2. The majority of needling procedures and incisional bleb revisions was performed within the first 100 weeks (approximately 2 years) after XEN gel stent implantation. On average, incisional bleb revisions were necessary later in the follow-up period than needling procedures Table 2.

The mean interval between the first needling procedure and a subsequent incisional bleb revision was 31.8 ± 51.7 weeks (median: 7 weeks, range: 0-263 weeks), calculated in eyes that underwent both procedures in that sequence. Conversely, the mean interval between the first incisional bleb revision and a subsequent needling procedure was 22.2 ± 33.3 weeks (median: 10 weeks, range: 1-90.8 weeks).

**Table 2:** Frequency and timing of needling procedure and incisional bleb revision procedures after XEN gel stent implantation

Parameter	Needling procedure	Incisional bleb revision
Eyes undergoing ≥1 procedure, n (%)	417 (53.9)	137 (17.8)
Mean number per eye	0.86 ± 0.99	0.20 ± 0.46
Mean time to first procedure (weeks ± SD)	33.6 ± 49.1	60.9 ± 62.5
Mean time to second procedure (weeks ± SD)	50.9 ± 59.5	51.5 ± 66.7
Mean time to third procedure (weeks ± SD)	81.3 ± 68.8	51.3 ± 46.7
Mean interval between first needling procedure and subsequent incisional bleb revision (weeks ± SD), median	31.8 ± 51.7, median: 7	
Mean interval between first incisional bleb revision and subsequent needling procedure (weeks ± SD), median	22.2 ± 33.3, median: 10	

### Use of antimetabolites in revision surgery

In the first needling procedures, 5-FU was applied in 161 cases (38,6%) and MMC was used in 57 cases (13.7%), whereas no antimetabolites were used in 199 needling procedures (47,7%). Regarding second needling procedures, 5-FU was used in 114 (65.5%) and MMC in 25 eyes (14.4%), and no antimetabolites were applied in 35 cases (20.1%).

In first incisional bleb revisions, 5-FU was used in 60 (45.5%) and MMC in 23 (17.4%), and no antimetabolites in 49 cases (37.1%). In second incisional bleb revisions, 5-FU was applied in 6 (46.2%), MMC in 2 (15.4%), and no antimetabolites in 5 eyes (38.5%).

## Discussion

In this large retrospective single-center cohort study, we evaluated the incidence, timing and determining risk factors of postoperative needling procedure and incisional bleb revision following XEN gel stent implantation in eyes with primary and secondary glaucoma having partly received glaucoma surgeries before. The analysis revealed several findings of interest. First, more than half of all eyes required at least one needling procedure, and nearly one in five underwent an incisional bleb revision during long-term follow-up. Second, the majority of bleb interventions was performed within the first 100 weeks after implantation, although the broad range of time intervals shows considerable interindividual variation in the scarring response. Third, while the risk of requiring needling procedures was not significantly associated with any of the examined factors, male sex and phakic lens status were independently associated with a higher hazard of incisional bleb revision.

Reported rates of needling procedures after XEN gel stent implantation vary widely across studies. For instance, after two years, needling rates of 28.4% [9], 34.5% [10], 37.7% [11], 41.1% [12] and 45% [13] were reported. After three years, needling rates in the literature vary between 28.5% [14], 42.9% [15], and 43.0% [16]. Steiner et al. described a general needling procedure and incisional bleb revision rate of 49.6% without specifying a mean follow-up period [17]. A needling rate of 42% has been observed in a four-year follow-up in a Swedish population, but with the majority of needling procedures occurring in the first six months after implantation [18]. Our observed needling rate of 53.9% is higher. However, our follow-up period is also longer, extending up to 90 months. Even though most needling occurs in the first 100 weeks, i.e., approximately 2 years, after implantation, the range of needling procedure time points is still high, so that late needling procedures due to the longer follow-up period could potentially explain the higher needling rate in our study. Furthermore, we advocate the therapeutic approach of performing bleb intervention at an early stage in the case of rising IOP values during the postoperative period, rather than reapplying IOP-lowering medication, probably leading to higher revision rates. Differences in study populations, such as glaucoma subtypes or previous surgeries, may also explain differences in needling rates.

Regarding incisional bleb revisions, rates between 3.9% [18],17.5% after four years [19], respectively, are described in the literature. A rate of incisional bleb revisions of 18.8% after three years has been reported [20]. The frequency of incisional bleb revision in our cohort (17.8%) aligns with these findings. In a study by Widder et al., in which the authors advocate for incisional bleb revision over needling procedure, the revision rate was 34% after 18-months follow-up [21]. Another study reported a rate of 33.3% after a mean follow-up of 330 days [22]. Differences in follow-up duration, patient selection and differing treatment approaches in cases of bleb scarring are possible factors explaining different rates of revision surgery. There is a lack of studies describing incisional bleb revision after longer follow-up periods than 4 years. Our extended observational period of up to 90 months allowed later-occurring fibrosis to be captured.

In the current analysis, mean ± SD time to first needling procedure was 33.6 ± 49.1 weeks. Previous studies report shorter mean times to first needling procedure of 4.7 ± 3.0 months (∼20.4 ± 13.0 weeks) [23] or 152 ± 160 days (21.7 ± 22.9 weeks) [12]. A comprehensive review describes a median time to first needling procedure of 59.5 days (8,5 weeks) with a range of 6–582 days (0.9. − 83.1 weeks). In our sample, the mean time to first needling procedure was longer, although the median was substantially shorter (14 weeks in the current study), indicating that many needling procedures occurred early while a smaller proportion took place much later. This pattern suggests the presence of one subgroup of eyes with early fibrotic changes and another in which late-onset bleb dysfunction develops years after implantation. Only few studies have systematically assessed such late needling events; our findings provide real-world evidence that bleb failure is not confined to the early postoperative period.

In our cohort, the mean time to first incisional bleb revision was 60.9 ± 62.5 weeks (median: 37 weeks). Compared with existing literature, our mean interval is longer: Evers et al. described a mean of 330 days (47.1 weeks) and a median of 70 days (10 weeks; range 39–1624 days) to the first open bleb revision [22], while Theilig et al. reported a shorter mean interval of 170.3 ± 265.6 days (24.3 ± 38.0 weeks) [24]. Whether this reflects a genuinely later development of clinically significant fibrosis in our population or is instead influenced by our clinical decision-making, where needling was often attempted before proceeding to incisional revision, cannot be definitively determined.

Reitsamer et al. observed a mean of 1.6 needling procedures per eye at 24 months, which exceeds the rate in our cohort (0.86 (± 0.99 SD) over the entire follow-up) [12]. In contrast, a recent meta-analysis reported a mean of only 0.38 needling procedures per patient [25]. Taken together, the mean number of needling procedures in our cohort lies between previously reported rates. Differences between studies may be explained by variations in postoperative management and the threshold of performing a needling procedure.

### Interpretation of risk factors

A key finding of this study is that male sex and phakic lens status were independently associated with a significantly increased hazard of incisional bleb revision. While the influence of sex on outcomes after filtering surgery has been examined in some other contexts, evidence remains mixed. Steiner et al. found male sex to be a risk factor for failure after XEN45 implantation [17]. Also, Gillmann et al. reported male sex as a strong predictor of failure after XEN implantation in POAG but not in pseudoexfoliative glaucoma [26]. In contrast, a recent study from Tan et al. examining risk factors for failure in XEN gel stent and trabeculectomy did not find any significant associations for male sex or lens status [27], and a study analysing risk factors in a 20-year follow-up after trabeculectomy did not find any sex-dependent associations [28]. Contrary to this, a retrospective study revealed male gender as a potential risk factor for reduced success rates after trabeculectomy [29]. Sex- and hormone-dependent differences in wound-healing biology have been extensively described in the literature and are known to vary across tissues [30, 31]; such mechanisms may help explain why male patients in our cohort appeared more prone to exuberant bleb fibrosis and consequently required incisional bleb revision more frequently. However, the underlying biologic mechanisms remain to be clarified.

In the current analysis, phakic lens status was significantly associated with higher risk of incisional bleb revision. In an earlier study conducted by our working group, we also observed a significantly higher rate of incisional bleb revisions in phakic patients than in pseudophakic eyes with pseudoexfoliative glaucoma following XEN implantation [32]. Notably, Buffault et al. reported a statistically lower needling procedure rate in phakic eyes compared to pseudophakic eyes after XEN implantation [33], and other studies observed no differences in bleb revision rates between phakic and pseudophakic eyes with XEN gel stents [34, 35]. The reasons for the higher rate of incisional bleb revision in phakic eyes in our study cohort remain uncertain, but several mechanisms may contribute. Pseudophakic eyes exhibit a more stable anterior chamber configuration, with reduced fluctuations in anterior chamber depth and less dynamic movement of the lens–iris diaphragm [36, 37]. Such anatomical stability may contribute to more consistent aqueous flow through the implant and might decrease the tendency toward subconjunctival fibrosis. These mechanisms could, at least in part, explain the lower revision rates observed in pseudophakic eyes.

Interestingly, none of the examined baseline parameters, including age, baseline IOP, postoperative day 1 IOP, and prior glaucoma surgery, were significantly associated with the need for needling procedure. This might hint at the heterogeneity of early bleb healing. Possibly, it is influenced predominantly by local tissue factors that are not captured by routine clinical variable, e.g. stent position relative to Tenon’s capsule or subtle inflammatory variations.

### Clinical implications and future directions

Our data suggest several clinical implications. The high cumulative rate of postoperative bleb intervention demonstrates the necessity of close monitoring especially throughout the first two postoperative years, with particular attention to male and phakic patients, who might be at higher risk for incisional bleb revision. However, the delayed occurrence of some bleb fibroses also warrants long-term follow-up rather than limiting surveillance to the early postoperative period.

Future studies prospectively evaluating modifiable predictors of bleb failure and examining different antimetabolite dosing for needling procedures and incisional revisions, can help further optimize surgical long-term outcomes. Comparative studies between XEN gel stent, Preserflo Microshunt, and trabeculectomy may help clarify the role of each procedure within the spectrum of subconjunctival filtration surgery.

### Strengths and limitations

Strengths of this study include its large study population size – the largest single-center cohort of XEN gel stent patients reported to date – its long follow-up duration, and the consistency of surgical technique and postoperative management afforded by having all procedures performed by a single experienced surgeon. This reduces variability and strengthens internal validity.

Limitations that warrant consideration are the retrospective design with the inherent risks of bias. The decision to perform a needling procedure or incisional revision was at the surgeon’s discretion and not standardized by a protocol, potentially influencing timing and frequency. Variations in the use of antimetabolites were based on clinical judgement and may have affected outcomes. Our heterogeneous patient population, while reflecting real-world practice, leads to variability in disease severity and scarring potential. We did not perform glaucoma subtype-stratified or subtype-adjusted analyses for needling procedures and incisional bleb revision; therefore, potential subtype-specific differences cannot be excluded. However, this case mix reflects routine clinical practice, where XEN is frequently used in complex eyes, and therefore informs real-world revision patterns. Finally, the study population consisted predominantly of Caucasian patients; given that fibrotic responses have been shown to differ across ethnicities [38], the generalizability of our findings may be limited. Future research should therefore include larger cohorts from underrepresented populations to validate and expand upon these results.

## Conclusion

In this large single-center cohort, more than half of all eyes required a postoperative needling procedure and nearly one in five underwent incisional bleb revision in the long-term follow-up up to 90 months (7.5 years). Bleb revisions occurred most commonly within the first two years after XEN gel stent implantation. Male sex and phakic lens status were independently associated with a higher risk of incisional revision, whereas none of the examined baseline factors predicted the need for a needling procedure. Understanding which patients are at higher risk for bleb failure can improve surgical planning and patient counselling and can support individualized management strategies.

## Data Availability

The data that support the findings of this study are available from the corresponding author upon reasonable request. The data are not publicly available due to privacy and ethical restrictions.
